# Changes of Keratinized Mucosa Width Around Posterior Implants: A Retrospective Cohort Study

**DOI:** 10.1155/ijod/5801279

**Published:** 2026-04-24

**Authors:** Ziyao Han, Yangeng Xu, Tao Xu, Cui Wang, Yiping Wei, Wenjie Hu, Kwok-Hung Chung, Yunsong Liu

**Affiliations:** ^1^ Department of Periodontology, Peking University School and Hospital of Stomatology & National Center of Stomatology & National Clinical Research Center for Oral Diseases & National Engineering Research Center of Oral Biomaterials and Digital Medical Devices, Beijing, China; ^2^ Department of Emergency, Peking University School and Hospital of Stomatology & National Center of Stomatology & National Clinical Research Center for Oral Diseases & National Engineering Research Center of Oral Biomaterials and Digital Medical Devices, Beijing, China; ^3^ NHC Key Laboratory of Digital Stomatology, Beijing, China; ^4^ Department of Restorative Dentistry, University of Washington, Seattle, Washington, USA, washington.edu; ^5^ Department of Prosthodontics, Peking University School and Hospital of Stomatology & National Center of Stomatology & National Clinical Research Center for Oral Diseases & National Engineering Research Center of Oral Biomaterials and Digital Medical Devices, Beijing, China

**Keywords:** dental implant, keratinized mucosa, soft tissue assessment

## Abstract

**Objective:**

To investigate the changes in keratinized mucosa width (KMW) at posterior implant sites through implant treatment procedures.

**Materials and Methods:**

In this retrospective study, we measured the buccal KMW at three time points: immediately before implant placement (T0), before definitive prosthesis delivery (T1), and within 1 month after prosthesis delivery (T2). We analyzed changes in the KMW at the implant sites between these time points, using both submerged and non‐submerged techniques.

**Results:**

A total of 72 patients and 98 implants were included. The KMW changed significantly from T0 to T1 (1.33 mm, 95% confidence interval [CI] [1.072, 1.561], *p* < 0.001) and from T0 to T2 (2.17 mm, 95% CI [1.929, 2.418], *p* < 0.001). No statistically significant differences were found in KMW changes between submerged and non‐submerged implants from T0 to T1 (*p* = 0.556) or from T0 to T2 (*p* = 0.572). The height of the abutment was significantly related to the changes in KMW from T0 to T1 (−0.274 mm, 95% CI [−0.544, −0.004], *p* < 0.005), but implant technique, alveolar ridge preservation (ARP), and the diameter of the abutment were not (*p* > 0.05).

**Conclusion:**

The KMW at posterior implant sites decreased significantly through implant treatment procedures, which were related to the height of abutment, but not to implant technique, history of ARP, or the diameter of abutment.

## 1. Introduction

The importance of keratinized mucosa width (KMW) has been examined and deemed essential in recent years for peri‐implant tissue health [1–7]. A minimum of 2 mm of KMW around dental implants is widely regarded as adequate, and 2 mm is routinely used as the cutoff value in both clinical practice and research [8, 9]. A meta‐analysis indicated that inadequate KMW (<2 mm) is associated with an increased risk of peri‐implant diseases and reduced tissue stability, and soft tissue augmentation procedures are recommended in such clinical scenarios [4]. Therefore, clinicians should carefully avoid any unnecessary damage to the KM, especially at the buccal aspect, during surgery to ensure at least 2 mm of KMW remains after prosthesis delivery [10–13].

Despite these precautions, some reduction in KMW is inevitable due to postoperative wound healing and the shift of the mucosal margin after abutment connection or crown insertion, with an average reduction ranging from 1.36 to 1.86 mm [14–17]. Other studies reported that the reduction of KMW during implant treatment may be influenced by surgical factors such as the submerged versus non‐submerged healing approaches and the timing of implant placement [14, 17].

Although this reduction was recognized, current evidence remains limited regarding longitudinal data on KMW changes at posterior implant sites from preimplantation through definitive prosthesis delivery, as well as the influencing factors, such as implant technique and abutment parameters, particularly for implant sites with submerged healing. Therefore, the primary aim of our present study is to evaluate changes in KMW at posterior implant sites from implant placement to definitive prosthesis delivery. Our null hypothesis was that no significant reduction in KMW would occur over this period.

## 2. Material and Methods

The present study was designed as a retrospective cohort study and was in accordance with the Helsinki Declaration revised in 2013. The approval of the present study was signed by the Institutional Review Board of Peking University School and Hospital of Stomatology (Number PKUSSIRB‐202058143) in December 2020, and the validity was extended in September 2022. The STROBE guidelines were followed for the report of the study.

### 2.1. Study Population and Selection

Patients who attended the Department of Periodontology, Peking University School, and Hospital of Stomatology and received implant treatment in the posterior regions from January 2015 to December 2020 were enrolled. All the included subjects had signed an informed consent. The inclusion criteria were as follows: (1) at least one implant located in the posterior region; and (2) clinical periodontal health according to the consensus reported by Chapple et al. [18]. Subjects who met the following criteria were excluded: (1) history of soft tissue augmentation at the implant site before or during implant treatment; (2) history of using medications which could increase the risk of gingival enlargement or affect soft tissue healing (such as nifedipine, cyclosporin, dilantin); (3) uncontrolled systemic diseases; (4) history of bisphosphonate use or head and neck radiation therapy; and (5) missing records of KMW measured from implant placement to prosthesis delivery.

### 2.2. Implant Therapy Protocols

All the implant surgeries were performed by an experienced periodontist (WH). Bone‐level implants (Straumann AG, Waldenburg, Switzerland) were inserted following the manufacturer’s instructions. Healing abutments were attached at the time of implant placement when the insertion torque exceeded 35 Ncm, and additional bone augmentation procedures were not required (such as the use of autogenous bone chips gathered during the surgery). We used single interrupted sutures (Monocryl 5/0, Ethicon) to close the wounds. For the submerged implants, second‐stage surgeries were performed after a 6‐month period. Following these surgical procedures, the fabrication of the definitive prostheses began 1 month later and was completed within 4 weeks. In the case of non‐submerged implants, the fabrication of the definitive prostheses occurred 6 months postimplant placement.

### 2.3. Clinical Measurements

As illustrated in Figure 1a, the KMW at the buccal aspect was measured with a periodontal probe (UNC‐15 periodontal probe; Hu‐Friedy, Chicago, IL) from the center of the planned implant position to the buccal mucogingival junction (MGJ) immediately before implant placement (T0) with an accuracy of 1 mm, and all measurements were recorded to the nearest 0.5 mm increment. The use of a surgical guide ensured the consistency between the planned and the actual implant positions [19]. Measurements of the KMW were taken before the delivery of the definitive prosthesis (at the time of impression‐making for prosthesis fabrication, T1) and within 1 month after definitive prosthesis delivery (T2). These measurements, taken from the mid‐buccal mucosal margin of the abutment/crown to the MGJ, were determined by assessing the mobility difference between the KM and the alveolar mucosa, which was accomplished by pushing the alveolar mucosa coronally (Figure 1).

Figure 1Measurements of keratinized mucosa width (yellow double‐headed arrow line) at (a) the time of impression‐making for definitive prosthesis fabrication, T1; (b) within 1 month after definitive prosthesis delivery, T2.

**Figure:**
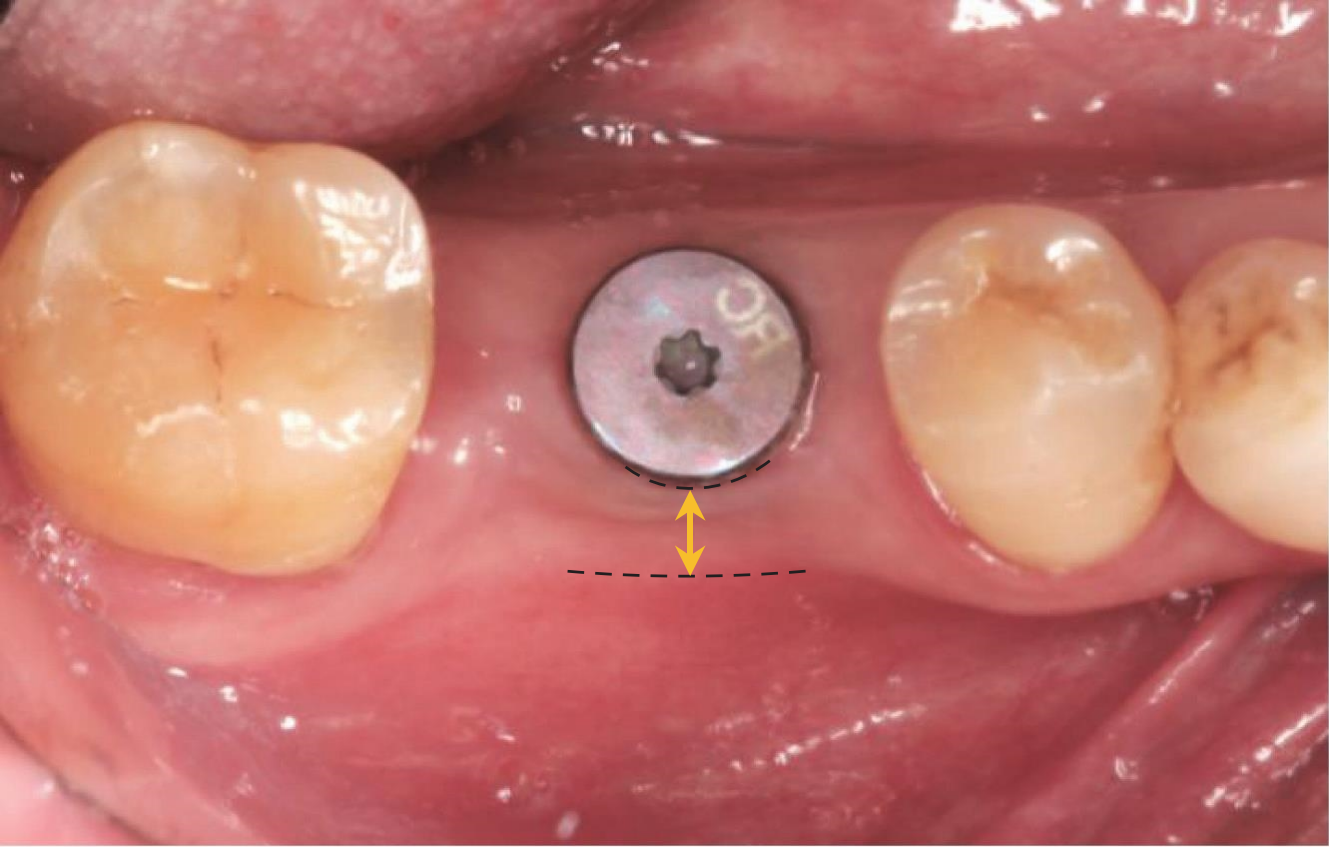
(a)

**Figure:**
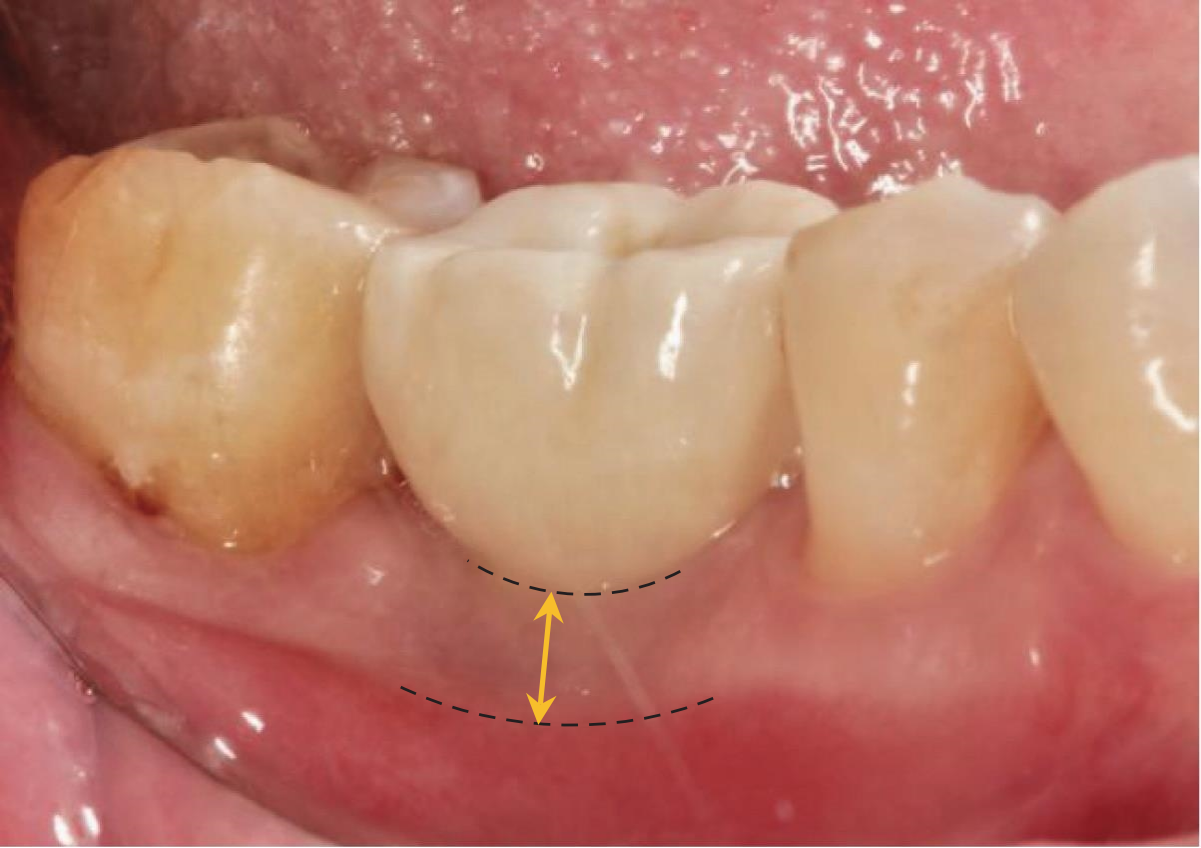
(b)

### 2.4. Sample Size Calculation

The sample size was calculated based on the width of the KM (i.e., primary outcome variable). Samples of at least 94 implants were necessary to detect a difference ≥0.4 mm in KMW, assuming a common standard deviation of 0.8 [14]. This calculation assumes an alpha error of 0.05 and a beta error of 0.2 in a bilateral contrast.

### 2.5. Intra‐Examiner Reliability

The measurements of KMW at different time points were all performed by a calibrated examiner (WH). The intraclass correlation coefficient (ICC) and Cohen’s kappa value were 0.958 (95% confidence interval [CI] [0.840, 0.989]) and 0.910, respectively.

### 2.6. Statistical Analysis

The primary outcome of the present study was the changes of KMW between the time of implant placement (T0), impression taking for definitive prosthesis fabrication (T1), and within 1 month after loading (T2). Descriptive statistics were presented using mean and standard deviation for continuous variables and frequency distribution for qualitative variables. All data were analyzed at the implant level. The normality was confirmed using Shapiro–Wilk tests.

In order to analyze the longitudinal changes in KMW at three time points, the linear mixed‐effects model (LMM) was used to account for the hierarchical structure of the data (multiple implants per patient and repeated measurements over time). The time point was modeled as a categorical fixed effect, with T0 set as the reference level. The model was adjusted for the following covariates: baseline, age, gender, implant position, history of alveolar ridge preservation (ARP), abutment parameters, and implant technique. To account for the nonindependence of observations, random intercepts for the patient and the implant (nested within patient) were specified in the model. An unstructured covariance structure was specified for the repeated measures. The significance of the fixed effects was tested using Type III ANOVA with Kenward–Roger degrees of freedom, which is robust for small samples, unbalanced data, and hierarchies. To directly compare the impact of implant technique on KMW change, ΔT0–T1 and ΔT0–T2 were calculated per implant and analyzed as the dependent variables in separate LMMs, with technique as the main fixed effect. Each model was adjusted for the same set of covariates above and included a patient‐level random intercept to account for within‐patient clustering.

Generalized estimating equation (GEE) analysis was used to explore the potential associations between changes of KMW (ΔT0–T1 and ΔT0–T2) and the implant technique, history of ARP, and the diameter and height of the abutment. The implant technique and the history of ARP were categorical variables, and the diameter and height of the abutment were continuous variables. All statistical analyses were performed with SPSS 26 (IBM Corporation, Armonk, NY, USA). The significance level was set at 95%.

## 3. Results

### 3.1. Data Set

Following preliminary screening, 79 patients initially met the inclusion criteria. However, seven of these patients were subsequently excluded due to incomplete records, specifically missing measurements of KMW taken either before implant placement or during impression‐making for definitive prosthesis fabrication. Therefore, the final study group consisted of 72 patients: 38 males and 34 females, ranging in age from 27 to 79 years, with an average age of 50.5 years. Ninety‐eight implants were placed in those patients. Most implants (76.6%) were placed at molar sites, and ARP was performed before implant placement in 40 sites (40.8%). Parameters of the healing abutments were also presented in Table 1.

**Table 1:** Demographics of implants.

Variable	*n* (%)
Implant position	
Upper premolar	14 (14.3)
Upper molar	32 (32.7)
Lower premolar	9 (9.2)
Lower molar	43 (43.9)
Implant technique	
Non‐submerged	29 (29.6)
Submerged	69 (70.4)
ARP	
Yes	40 (40.8)
No	58 (59.2)
Diameter of healing abutment	
3.6	7 (7.1)
4.5	38 (38.8)
5.0	9 (9.2)
6.0	44 (44.9)
Height of healing abutment	
4	36 (36.7)
5	8 (8.2)
6	54 (55.1)

*Note: n*, sample number.

Abbreviation: ARP, alveolar ridge preservation.

**Figure 2:**
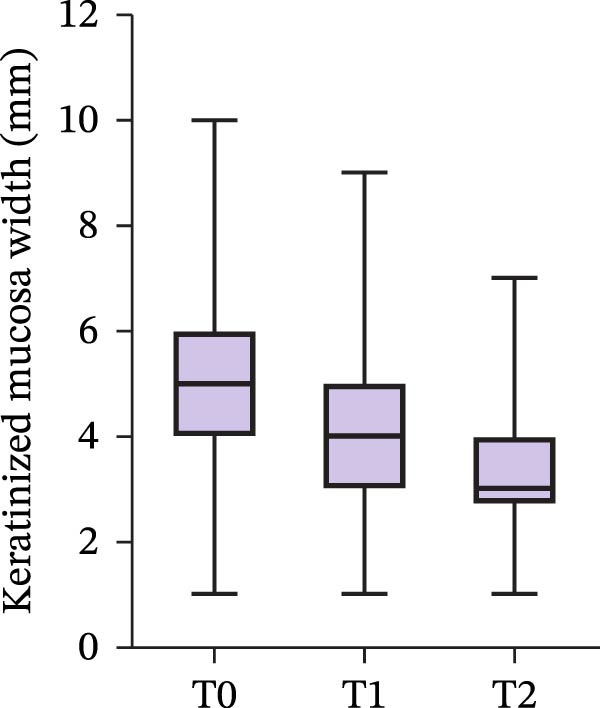
The overall changes in keratinized mucosa width at posterior implant sites. T0: immediately before implantation; T1: immediately before the impression taking for definitive prosthesis fabrication; T2: within 1 month after loading. Sample sizes at all time points were *n* = 98.

Changes of KMW around implants from T0 to T2 are presented in Figure 2. The mean KMWs around 98 implants were 5.54 ± 0.18 mm, 4.22 ± 0.150 mm, and 3.37 ± 0.13 mm at T0, T1, and T2 (Table 2). Based on the results from the LMM, we identified significant changes in KMW from T0 to T1 and from T0 to T2. These changes amounted to 1.32 mm (95%CI [1.07, 1.56], *p* < 0.001) and 2.17 mm (95%CI [1.93, 2.42], *p* < 0.001) respectively.

**Table 2:** Changes in keratinized mucosa width at posterior implant sites between different time points.

Keratinized mucosa width mean ± SD (mm)	KMW mean ± SD (mm)	Coefficient (mm) (95% CIs: lower, upper)	*t* (df)	*p*‐Value
T0	5.54 ± 0.18	Ref.	—	—
T1	4.22 ± 0.15	−1.32 (−1.561, −1.072)	*t* (223.34) = −8.67	<0.001^∗∗^
T2	3.37 ± 0.13	−2.17 (−2.418, −1.929)	*t* (223.34) = −14.31	<0.001^∗∗^

*Note:* Results of the linear mixed‐effect model, in which the fixed effect was time points with adjustment for age, gender, history of alveolar ridge preservation, the abutment parameters, and the implant technique. The patient and implant were considered as random effects. T0, immediately before implantation; T1, immediately before the impression taking for definitive prosthesis fabrication; T2, within 1 month after loading. Coefficients represent the mean change of KMW from T0 to that specific time point.

Abbreviations: CIs, confidence intervals; df, degrees of freedom; SE, standard error.

^∗∗^ *p* < 0.001.

Changes in KMW around implants placed with non‐submerged and submerged technique from T0 to T1 (*p* = 0.437) and T0 to T2 (*p* = 0.811) showed no statistically significant differences (Supporting Information 1: Table S1).

GEE was employed to evaluate factors associated with changes in KMW at posterior implant sites. Potential interaction effects among the factors were tested first, and none reached statistical significance (*p* > 0.05, Supporting Information 2: Table S2). Results from the GEE analysis showed that a 1‐mm increment in abutment height was associated with a 0.274‐mm KMW loss from T0 to T1 (95% CI [−0.544, −0.004], *p* = 0.047). Other factors, such as the implant technique, ARP, and the diameter of the abutment, did not significantly impact the changes in KMW around implants from either T0 to T1 or from T0 to T2 (*p* > 0.05, Table 3).

**Table 3:** Generalized estimating equation analysis of factors associated with the keratinized mucosa width at posterior implant sites.

	ΔT0–T1			ΔT0–T2		
Variables	Wald^2^	B (95%CI: Lower, Upper)	*p*‐Value	Wald^2^	B (95%CI: lower, upper)	*p*‐Value
Intercept	0.081	0.319 (−1.874, 2.511)	0.776	0.023	−0.203 (−2.167, 2.815)	0.880
Implant technique	0.580	—	—	0.022	—	—
Non‐submerged	Reference			Reference		
Submerged	—	0.213 (−0.335, 0.761)	0.446	—	−0.048 (−0.688, 0.592)	0.883
ARP	0.091	—	—	0.073	—	—
No	Reference			Reference		
Yes	—	−0.074 (−0.553, 0.406)	0.764	—	−0.073 (−0.604, 0.458)	0.787
Diameter of the abutment	0.210	−0.066 (−0.347, 0.216)	0.646	1.455	−0.202 (−0.529, 0.126)	0.228
Height of the abutment	3.946	−0.274 (−0.544, −0.004)	0.047^∗^	1.386	−0.169 (−0.450, 0.112)	0.239

*Note:* T0, immediately before implantation; T1, immediately before the impression taking for definitive prosthesis fabrication; T2, within 1 month after loading.

Abbreviation: ARP, alveolar ridge preservation.

^∗^ *p* < 0.050.

## 4. Discussion

Recently, there has been an increasing focus on the evaluation of peri‐implant soft tissue quantity and quality [20]. Current literature demonstrates that adequate KMW plays a protective role in maintaining peri‐implant health [4, 7]. Therefore, gaining insight into KMW alterations during implant treatment is important for predicting postloading KMW adequacy and determining the necessity of soft tissue augmentation, particularly for sites at high risk of KMW inadequacy [17]. This retrospective cohort study aimed to analyze the changes in KMW around posterior implants from the baseline (before implant placement) to definitive prosthesis delivery. Our findings reject the null hypothesis, revealing a significant reduction in KMW from T0 to T2. This study found an average KMW reduction of 2.17 mm at posterior implant sites from baseline to completion of implant reconstruction.

Few reports are available evaluating KMW changes at implant sites throughout the entire treatment period. Liu et al. [15] noted a KMW reduction of 1.65 ± 1.19 mm in implants immediately placed in compromised sockets postloading. Similarly, Cordaro et al. [14] reported a reduction of 1.86 ± 1.29 mm after immediate implant placement with submerged healing. The reduction of KMW could be attributed to the closure of the coronal advanced flap, possibly due to the coronal shift of the MGJ and suture tension [21]. A systematic review found comparable changes in hard and soft tissues between immediately placed implants and those placed using conventional protocols [22]. Wang et al. [23] reported a smaller KMW reduction of only 0.5 mm throughout the implant treatment, but the measurement of KMW was taken from the interproximal mucosal margin of the abutment to the MGJ. Thus, data from different studies are challenging to compare due to these variations. The KMW reduction in our study was higher than in previous reports, which could be due to variations in implant techniques and reference points for KMW measurements. It should be pointed out that in the present study, KMW measurements at T0 were based on the planned implant position, whereas those at T1 and T2 were referenced to the abutment margin of the definitive prosthesis. Despite the use of the surgical guide to minimize deviations between the actual and planned implant positions, the inconsistency in reference points nevertheless introduced a potential systematic measurement bias. Thus, the apparent reduction observed from T0 to T1 may not totally represent genuine biological recession of the KM but could also be partly attributable to a shift in the measurement reference point. Consequently, the interpretation of KMW changes requires particular caution. Measurements taken directly at the incision site in an investigation may provide more accurate data on biological changes [17].

Our study observed a decrease in KMW by 1.32 mm before the delivery of the crown, aligning with results from a prospective case series investigating changes in KMW around non‐submerged implants [17]. The cited study noted a KMW reduction of 1.39 ± 0.79 mm 3 months after non‐submerged healing. It suggested that an inadequate abutment height might lead to further loss of KMW. However, in our study, the height of the abutments was between 4 and 6 mm, which is higher than the mucosal thickness, thereby preventing the KM from overlapping the abutment. This study revealed that for each 1‐mm increase in abutment height, a corresponding 0.27‐mm reduction in KMW was observed from T0 to T1. There is limited evidence regarding the impact of abutment height on changes in KMW. This reduction could be explained by possible challenges in compromised oral hygiene around taller abutments, which may promote plaque accumulation and chronic inflammation, thereby endangering keratinized tissue stability [24]. Contributing factors might also include mechanical tension and microcirculatory compression‐induced ischemia [25]. However, the observed association was not directly supported by plaque indices, inflammation scores, or soft tissue thickness measurements, and further validation is required, given the limited height range and uneven sample distribution in the present study. Finally, this small reduction (≈0.27 mm) is of limited clinical relevance as it fell within the measurement error range of a periodontal probe. Therefore, abutment design is one of the many factors influencing peri‐implant soft tissue biology, but current evidence does not justify changing routine clinical selection of abutment height based on this finding alone.

Moreover, we identified a mean KMW decrease of 0.85 mm from T1 to T2, while another study discovered an average reduction of 0.86 ± 0.71 mm in KMW 3 months post‐abutment connection compared to immediate postoperative measurements [26]. This discrepancy might be due to different surgical techniques and variations in the methodologies and timelines for evaluating KMW. Kim et al. [26] analyzed KMW at the mesial, middle, and distal sites, using the average value, while our study and others focused only on the mid‐facial KMW [14, 15, 17].

It was demonstrated that an extended duration of functional loading correlates with a higher risk of inadequate peri‐implant KMW [27]. Investigations focused on peri‐implant KMW changes subsequent to functional loading have generated disparate findings [28, 29]. A mean KMW reduction of 1.08 ± 1.31 mm was observed at immediately placed implants during the first year after loading [28]. Conversely, another study reported a slight increase in KMW around non‐submerged implants (0.47 ± 0.38 mm) following 12 months of loading [29]. In light of the crucial role of preserving adequate KMW for maintaining peri‐implant tissue health, the preoperative KMW assessment should consider possible reduction during implant treatment. Soft tissue augmentation should be performed at sites with a higher risk of inadequate peri‐implant KMW [7]. A sizable proportion (41.9%) of molar implant sites exhibited less than 4 mm of KMW before implant placement [30]. Given the likelihood of KM loss during implant treatment, soft tissue augmentation is recommended to preserve adequate KM around the implant restoration [30, 31]. In the present investigation, only 1 implant showed KMW < 2 mm during follow‐up; the low rate is likely related to the generally good baseline soft tissue condition (73.5% of sites had KMW >4 mm at T0, max 10 mm). These observations imply that for posterior implant sites with initially sufficient KMW (>4 mm), preventive soft tissue grafting may not be routinely required. Clinical decisions should therefore focus more on evaluating the initial tissue characteristics. We present a typical case with a preimplantation KMW of 2 mm that underwent a free gingival graft (FGG) (Figure 3). After delivering the prosthesis, the buccal aspect of the peri‐implant mucosa was predominantly covered by the grafted KM (Figure 3d).

Figure 3A typical case presenting the reduction of keratinized mucosa width after implant treatment. (a) missing lower molars presenting with 2 mm of buccal keratinized mucosa, the mucogingival junction was marked with a dotted line; (b) immediately and (c) 2 months after receiving free gingival graft, the border of the original and grafted keratinized mucosa was distinguishable, marked with a dotted line; (d) after definitive prosthesis delivery, the buccal peri‐implant mucosa consisted mostly of the grafted keratinized mucosa, the black arrows marked the mid‐buccal mucosal margin of the implant restorations.

**Figure:**
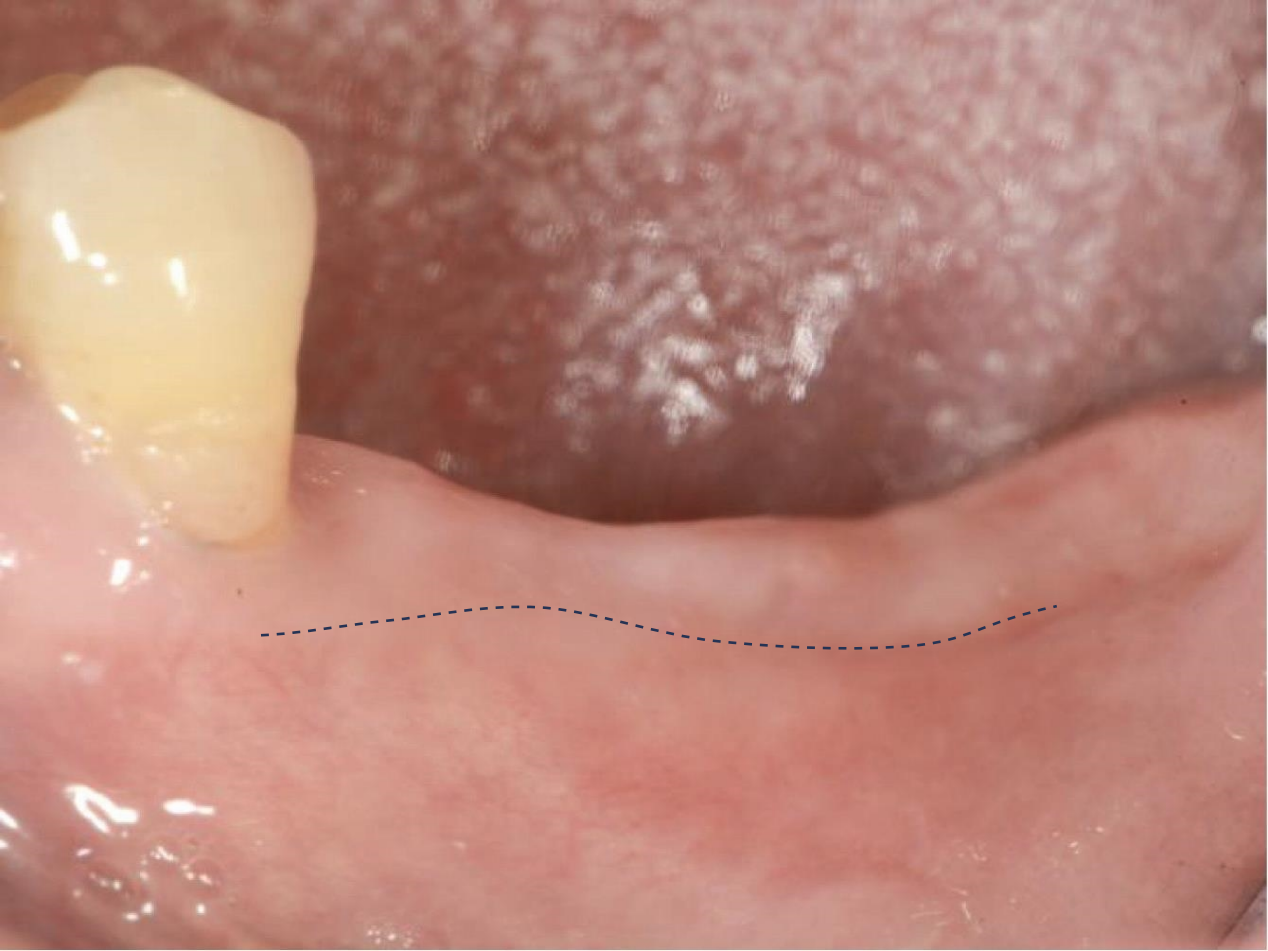
(a)

**Figure:**
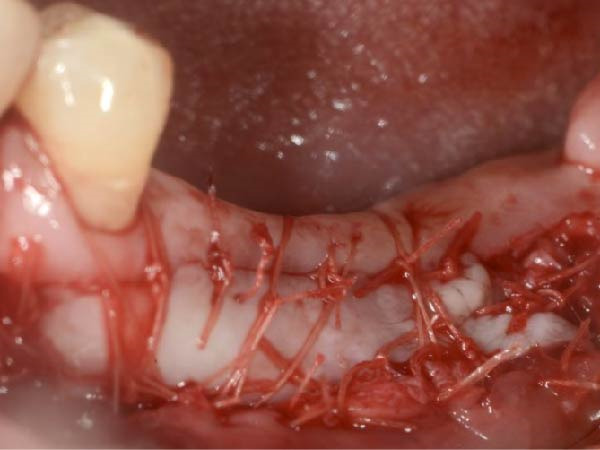
(b)

**Figure:**
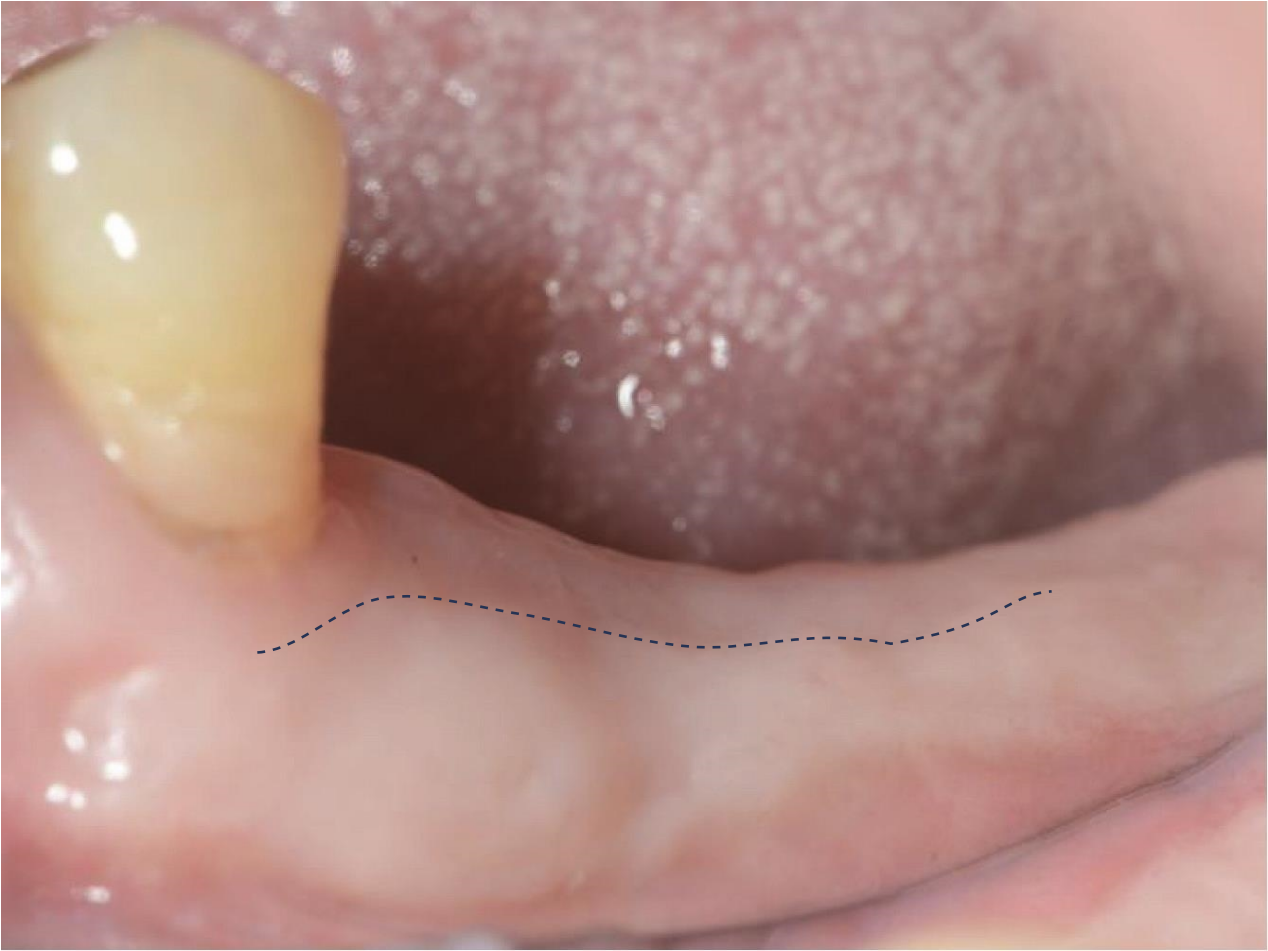
(c)

**Figure:**
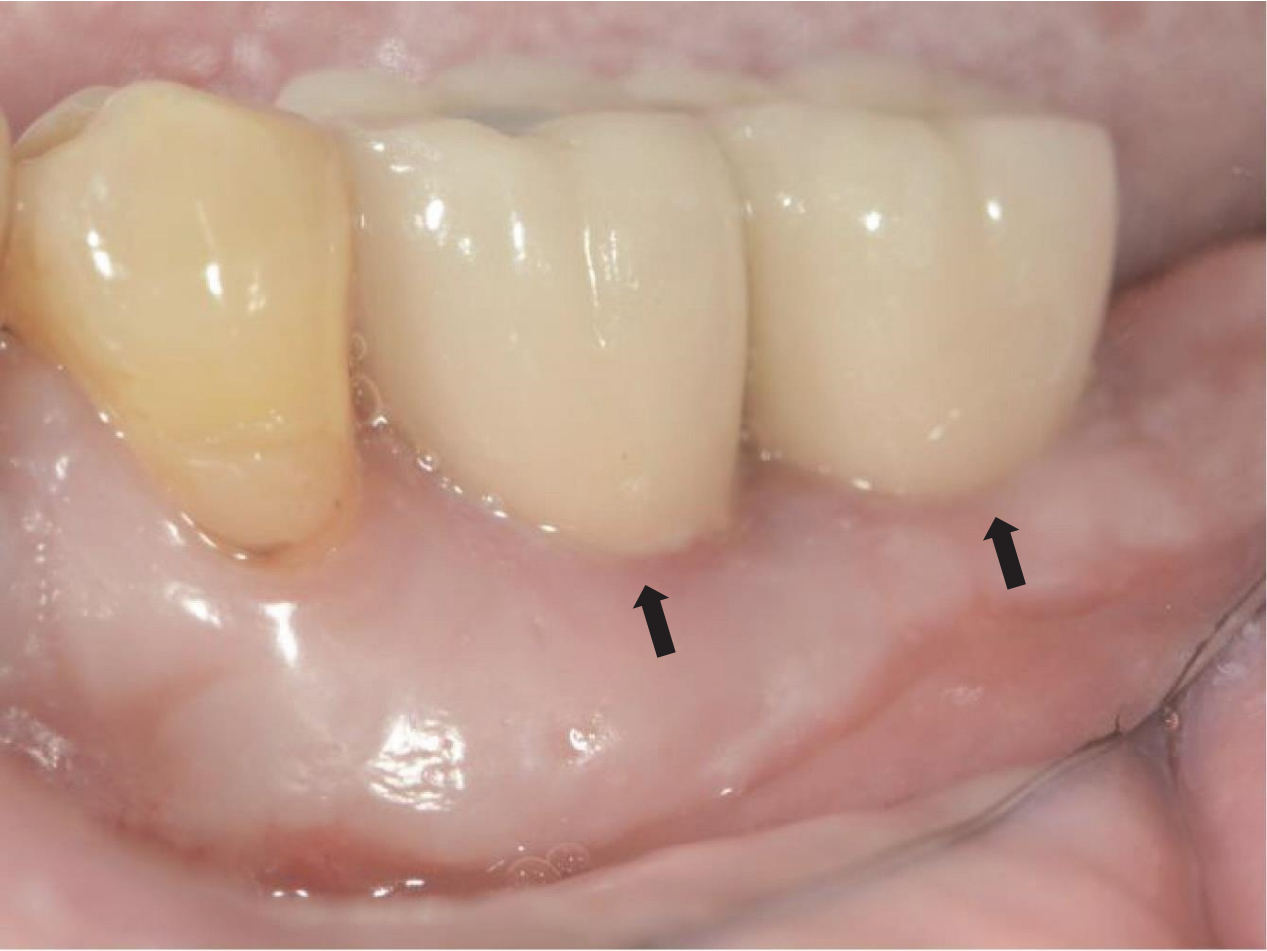
(d)

It should be acknowledged that the present study has certain limitations. First, due to the retrospective design, the sample size was constrained by the absence of some patients’ clinical records, which could have resulted in a larger sample size. Second, KMW was measured using a periodontal probe. Although the observed changes exceeded the measurement error, KMW could have been assessed more accurately and reliably with a stent and an intraoral scanner. The three‐dimensional data collected by the intraoral scanner are more precise and can facilitate measurements on a curved surface, ensuring repeatability at the same location [17, 32]. Third, the inconsistency in reference points for KMW measurements across time points may have introduced systematic measurement bias. Fourth, biological or functional outcome measurements were lacking. Moreover, a short post‐loading observation period might limit the assessment of long‐term stability. Finally, this study did not account for potential confounders such as soft tissue phenotype, flap design, or suturing details. The inability to adjust for these factors represents a limitation and a potential source of bias in the analysis. Hence, the results of this study should be interpreted cautiously within the specific clinical and technical context (the specific surgical technique, operators, and implant system) and may apply only to posterior implant sites. Future studies should adopt a prospective design and utilize more precise methods for evaluating KMW, with a larger sample size.

## 5. Conclusion

Given the limitations of the current study, we observed an average reduction of 2.17 mm in KMW at posterior implant sites from the time before implant placement to definitive prosthesis delivery. Presurgical soft tissue evaluations should take into account the potential loss of KM and the potential demand for soft tissue augmentation procedures.

## Author Contributions

Ziyao Han and Yangeng Xu contributed equally to the conceptualization, data curation, formal analysis, investigation and the writing, review and editing of the manuscript. Tao Xu contributed to the data curation, investigation, methodology, and the review and editing of the manuscript. Cui Wang and Yiping Wei contributed to the data curation, methodology and the review and editing of the manuscript. Wenjie Hu contributed to the conceptualization, data curation, funding acquisition, methodology, project administration and supervision and the review and editing of the manuscript. Kwok‐Hung Chung contributed to the conceptualization, investigation, validation and the review and editing of the manuscript. Yunsong Liu contributed to the investigation and the review and editing of the manuscript.

## Funding

The present study was supported in part by the National Natural Science Foundation of China (Grant 82173647), Capital’s Funds for Health Improvement and Research (Grant 2022‐2‐4103).

## Disclosure

All the authors approved the submission of the final versions of the manuscript.

## Conflicts of Interest

The authors declare no conflicts of interest.

## Supporting Information

Additional supporting information can be found online in the Supporting Information section.

## Supporting information


**Supporting Information 1** Table S1: Changes in keratinized mucosa width around implants placed with different techniques. Δ*T0-T1* and Δ*T0-T2* were calculated as the adjusted mean estimated based on independent linear mixed‐effects models. All models were adjusted for covariates and included patient‐level random intercepts. Abbreviations: T0, immediately before implantation; T1, immediately before the impression taking for definitive prosthesis fabrication; T2, within 1 month after loading; df, degrees of freedom.

**Supporting Information 2** Table S2: Interaction term test in factors associated with the keratinized mucosa width at posterior implant sites. Abbreviations: ARP, alveolar ridge preservation; T0, immediately before implantation; T1, immediately before the impression taking for definitive prosthesis fabrication; T2, within 1 month after loading; DA, Diameter of the abutment; HA, height of the abutment.

## Data Availability

The data that support the findings of this study are available from the corresponding author upon reasonable request.
