# Supplementary material for: Changes of Keratinized Mucosa Width Around Posterior Implants: A Retrospective Cohort Study
Source: Int J Dent. 2026 Apr 24;2026:5801279. doi: 10.1155/ijod/5801279 (PMC13108585; doi:10.1155/ijod/5801279)
Supplement: Supplementary file 1 — Supporting Information 1 Table S1: Changes in keratinized mucosa width around implants placed with different techniques. ΔT0-T1 and ΔT0-T2 were calculated as the adjusted mean estimated based on independent linear mixed‐effects models. All models were adjusted for covariates and included patient‐level random intercepts. Abbreviations: T0, immediately before implantation; T1, immediately before the impression taking for definitive prosthesis fabrication; T2, within 1 month after loading; df, degrees of freedom. [file IJOD-2026-5801279-s001.docx]

**Supplementary Table 1** Changes in keratinized mucosa width around implants placed with different techniques

|  | Implant technique | | t(df) | *p*-Value |
| --- | --- | --- | --- | --- |
|  | Non-submerged | Submerged |  |  |
| △T0-T1（mm） | -1.43 ± 0.26 | -1.20 ±0.16 | t(95.31)=0.458 | 0.437 |
| △T0-T2（mm） | -2.17 ± 0.26 | -2.10 ±0.16 | t(95.31)=0.466 | 0.811 |

△T0-T1 and △T0-T2 were calculated as the adjusted mean estimated based on independent linear mixed-effects models. All models were adjusted for covariates and included patient-level random intercepts.

Abbreviations: T0, immediately before implantation; T1, immediately before the impression taking for definitive prosthesis fabrication; T2, within 1 month after loading; df, degrees of freedom.
