# Supplementary material for: Changes of Keratinized Mucosa Width Around Posterior Implants: A Retrospective Cohort Study
Source: Int J Dent. 2026 Apr 24;2026:5801279. doi: 10.1155/ijod/5801279 (PMC13108585; doi:10.1155/ijod/5801279)
Supplement: Supplementary file 2 — Supporting Information 2 Table S2: Interaction term test in factors associated with the keratinized mucosa width at posterior implant sites. Abbreviations: ARP, alveolar ridge preservation; T0, immediately before implantation; T1, immediately before the impression taking for definitive prosthesis fabrication; T2, within 1 month after loading; DA, Diameter of the abutment; HA, height of the abutment. [file IJOD-2026-5801279-s002.docx]

**Supplementary Table 2** Interaction term test in factors associated with the keratinized mucosa width at posterior implant sites

|  | △T0-T1 | | △T0-T2 | |
| --- | --- | --- | --- | --- |
| Interaction term | *B* | *p*-Value | *B* | *p*-Value |
| Implant technique·ARP |  |  |  |  |
| Non-submerged*ARP (yes) | 0.325 | 0.452 | -1.856 | 0.193 |
| Submerged*ARP (yes) | -0.731 | 0.594 | -0.206 | 0.502 |
| Submerged*ARP (no) | -0.533 | 0.697 | -2.635 | 0.074 |
| Non-submerged*ARP (no) | Reference | | Reference | |
| Implant technique·DA·HA |  |  |  | 0.704 |
| Submerged*DA*HA | -0.077 | 0.148 | 0.077 | 0.148 |
| Non-submerged*DA*HA | Reference | |  |  |
| DA·HA | 0.088 | 0.058 | 0.011 | 0.671 |

Abbreviations: ARP, alveolar ridge preservation; T0, immediately before implantation; T1, immediately before the impression taking for definitive prosthesis fabrication; T2, within 1 month after loading; DA, Diameter of the abutment; HA, height of the abutment.
